# Pathogenic T-Cell Responses in Immune-Mediated Glomerulonephritis

**DOI:** 10.3390/cells11101625

**Published:** 2022-05-12

**Authors:** Alexandra Linke, Gisa Tiegs, Katrin Neumann

**Affiliations:** 1 Institute of Experimental Immunology and Hepatology, Center of Experimental Medicine, University Medical Center Hamburg-Eppendorf, Martinistrasse 52, 20246 Hamburg, Germany; alexandra.linke@web.de; 2 Hamburg Center for Translational Immunology, University Medical Center Hamburg-Eppendorf, Martinistrasse 52, 20246 Hamburg, Germany

**Keywords:** immune-mediated GN, kidney inflammation, crescent formation, renal Th1 and Th17 responses, cytotoxic CD8^+^ T cells, tissue-resident memory T cells

## Abstract

Glomerulonephritis (GN) comprises a group of immune-mediated kidney diseases affecting glomeruli and the tubulointerstitium. Glomerular crescent formation is a histopathological characteristic of severe forms of GN, also referred to as crescentic GN (cGN). Based on histological findings, cGN includes anti-neutrophil cytoplasmic antibody (ANCA)-associated GN, a severe form of ANCA-associated vasculitis, lupus nephritis associated with systemic lupus erythematosus, Goodpasture’s disease, and IgA nephropathy. The immunopathogenesis of cGN is associated with activation of CD4^+^ and CD8^+^ T cells, which particularly accumulate in the periglomerular and tubulointerstitial space but also infiltrate glomeruli. Clinical observations and functional studies in pre-clinical animal models provide evidence for a pathogenic role of Th1 and Th17 cell-mediated immune responses in cGN. Emerging evidence further argues that CD8^+^ T cells have a role in disease pathology and the mechanisms of activation and function of recently identified tissue-resident CD4^+^ and CD8^+^ T cells in cGN are currently under investigation. This review summarizes the mechanisms of pathogenic T-cell responses leading to glomerular damage and renal inflammation in cGN. Advanced knowledge of the underlying immune mechanisms involved with cGN will enable the identification of novel therapeutic targets for the replacement or reduction in standard immunosuppressive therapy or the treatment of refractory disease.

## 1. Introduction

Glomerulonephritis (GN) is an inflammatory kidney disease affecting the glomeruli and the tubulointerstitium, which may result in the reduction or even a loss of kidney function. When renal inflammation leads to the destruction of glomerular capillaries and the release of pro-inflammatory cytokines and chemokines into the Bowman’s space, parietal epithelial cells, lining the inner side of the Bowman’s capsule (BC) proliferate and build crescents [1,2]. This crescentic (c)GN, or rapidly progressive GN (RPGN), is the most severe form of GN, which frequently leads to end-stage renal disease within days to weeks. According to pathological findings, cGN is divided into three major forms, namely pauci-immune GN, as observed in anti-neutrophil cytoplasmic antibody (ANCA)-associated GN, characterized by scarcity or absence of immune complex deposition, anti-glomerular basement membrane (anti-GBM) GN or Goodpasture’s disease, in which the non-collagenous domain of α3 type IV collagen represents an autoantigen in the GBM, and immune complex-mediated GN as observed in lupus nephritis (LN) [1,3]. These (auto)inflammatory syndromes resulting in crescent formation have in common a striking accumulation of T cells and monocytes/macrophages in renal tissue. CD4^+^ and CD8^+^ T cells are present in kidney biopsies and seem to infiltrate the kidney in equal numbers [4,5]. T cells are mainly found in the periglomerular and tubulointerstitial space and are less abundant within the glomeruli [4,6]. Renal T-cell infiltration positively correlates with disease activity in patients with ANCA-associated GN and LN, as indicated by proteinuria, serum creatinine levels, and histological scores [7,8]. Hence, the crescent formation has been described as a consequence of an upstream immunopathogenesis involving a significant T-cell response that results in a cellular response of parietal epithelial cells with concomitant destruction of renal tissue [8]. Since functional studies with respect to the role of T-cell subsets in immune-mediated GN have mainly been undertaken in experimental models, this review focuses on the pathogenic function of CD4^+^ T helper (Th) cell subsets and CD8^+^ T cells in murine models of cGN and also refers to related clinical findings.

## 2. Immune-Mediated GN

**Figure 1:**
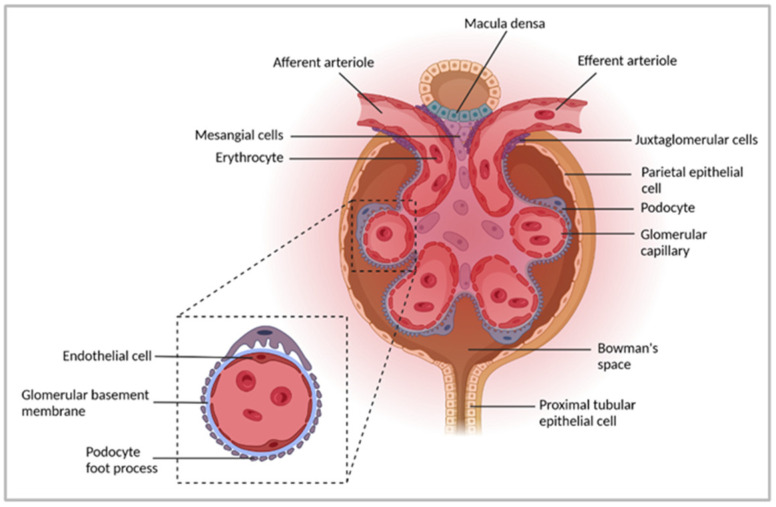
The functional unit of the kidney. The nephron, a functional unit of the kidney, consists of one glomerulus and the adjacent tubules. Blood enters the glomerulus through afferent arterioles, reaches glomerular capillaries for filtration, and exits the glomerulus via efferent arterioles. The glomerular filtration barrier consists of three layers comprising endothelial cells, the glomerular basement membrane, and podocytes. Mesangial cells are located within the capillary tuft. The internal surface of the BC is lined by parietal epithelial cells, which lead to the proximal convoluted tubule lined by proximal tubular epithelial cells. Fluids from glomerular capillaries are collected in the Bowman’s space and further processed within the tubular system to form the urine. Figure has been created with BioRender.com (accessed on 6 April 2022).

The kidneys are essential for maintaining homeostasis due to their ability to filter the blood, thereby removing waste products and metabolic and toxic substances. They reabsorb nutrients from the blood, maintain electrolyte, water, and acid–base balance, and regulate blood pressure and plasma osmolality. The functional unit of the kidney is the nephron, which consists of a renal corpuscle followed by a tubule. The nephrons filter the blood and produce urine. The corpuscle contains the filter consisting of the glomerulus, a capillary tuft surrounded by a filtration barrier, and the BC. The internal surface of the BC is lined by parietal epithelial cells. The filtration barrier, size and charge selective filter, entails vascular endothelial cells, the GBM, and podocytes. Within the glomerulus, mesangial cells are located inside the capillary tuft. Downstream of the glomerular filter, renal tubules are connected to the outer layer of the BC (Figure 1). The tubular system comprises a proximal tubule, the loop of Henle, and a distal tubule, which flows into collecting ducts and the renal calyces. The tubulointerstitium is located between the tubules and contains immune cells such as dendritic cells (DCs) and T cells, particularly under inflammatory conditions [9,10,11].

GN comprises a group of immune-mediated inflammatory kidney diseases that affect glomeruli, the surrounding tubulointerstitium, and tubuli. GN is one of the leading causes of chronic kidney disease and leads to an impairment of or even a loss of kidney function [1,12]. GN can develop in the course of a systemic disease, e.g., systemic lupus erythematosus (SLE) or vasculitis, with multiple symptoms or can present only renal findings. In histopathology, severe GNs are characterized by crescent formation as a result of destructive glomerular inflammation with the rupture of glomerular capillaries, leading to the release of cellular and humoral inflammatory mediators into Bowman’s space [12,13,14]. Especially the formation of crescents and breaches in BC quickly leads to an end-stage kidney disease, known as RPGN [14,15], which requires fast therapeutic intervention. At the moment, most GNs are treated with a combination of high-dose corticosteroids and immunosuppressive agents, such as cyclophosphamide, azathioprine, cyclosporine, or mycophenolate-mofetil to induce remission [16]. However, corticosteroids and other immunosuppressive agents are not specific for a particular GN, and many patients develop side effects. While some patients show recovery of kidney function after immunosuppressive treatment and dialysis, others do not respond and require kidney transplantation [10]. Consequently, more specific treatment options are needed for the different types of GN, necessitating a broader understanding of the underlying pathogenic immune-mediated mechanisms.

Clinically, GN can present as a nephrotic or nephritic syndrome or an overlap of both [1]. Nephrotic syndrome includes proteinuria with more than 3.5 g/1.73 m^2^ body surface within 24 h, leading to edema, hypoalbuminemia, and hyperlipidemia [17,18]. The loss of other blood proteins, including antithrombin III and immunoglobulins, further leads to thrombophilia [19] and a higher susceptibility to infections [18]. Nephritic syndrome comprises hematuria, edema, and arterial hypertension and is connected to acute renal inflammation [20]. Kidney biopsy is the gold standard for the definitive diagnosis of GN, allowing analysis by light microscopy, immunofluorescence, or electron microscopy. According to the pathology identified by these techniques, GN is divided into immune complex (IC), pauci-immune, and anti-GBM GN. IC-mediated GN, characterized by granular glomerular IC deposition, comprises four main GN diagnoses: LN, immunoglobulin A nephropathy (IgAN), membranoproliferative GN, and infection-associated GN [21]. Pauci-immune GN presents with proliferation and inflammation under light microscopy but does not or only minimally presents with IC deposition. Most patients with pauci-immune GN display diagnoses positive for ANCA, such as granulomatosis with polyangiitis, eosinophilic granulomatosis with polyangiitis, or microscopic polyangiitis [13,22,23]. The staining pattern of ANCA is either cytoplasmic (c-ANCA), associated with anti-proteinase 3 (PR3) antibodies, or perinuclear (p-ANCA), related to anti-myeloperoxidase (MPO) antibodies. MPO and PR3 are antigens mainly expressed by neutrophils and monocytes [23]. Anti-GBM disease, displaying linear staining for IgG along the glomerular capillary loops, is caused by the binding of anti-GBM antibodies to the non-collagenous domain 1 of the alpha 3 chain of type 4 collagen (α3(IV)NC1) [24].

## 3. Experimental Models to Study Pathogenic Mechanisms in Immune-Mediated GN

There are diverse experimental models of the different GN forms described, allowing investigation of disease-specific pathogenic mechanisms and identification of potential therapeutic targets. In transgenic mouse models of SLE, such as MRL/MpJ-*Fas^lpr^* (MRL-*lpr*) and NZB/NZW mice, and in inducible SLE models, e.g., induced by pristane treatment, mice develop a lupus-like disease with autoantibody production and LN [25]. The ddY mouse strain is a model of spontaneous IgAN that develops GN with mesangial IgA deposition accompanied by IgG, IgM, and C3 deposition [26,27], thereby also constituting a model of IC-mediated GN. A frequently used model of anti-GBM GN is the experimental autoimmune GN (EAG), induced by immunization of mice or rats with recombinant α3(IV)NC1, leading to the formation of anti-GBM antibodies [28,29]. Different murine models of autoimmune anti-MPO GN exist, either passively induced by transfer of anti-MPO antibodies or MPO-specific T cells from MPO-immunized mice or actively induced by immunization with MPO [30]. MPO-specific antibodies have been shown to activate neutrophils [31]. A subnephritogenic dose of anti-GBM antibodies is used to recruit neutrophils to the glomeruli, where they degranulate and deposit MPO. Glomerular MPO triggers a recall response, which is directed by the established anti-MPO autoimmunity. Pathological characteristics of this autoimmune GN model are proteinuria, focal, and segmental glomerulonecrosis [32,33]. Nephrotoxic nephritis (NTN) is the most widely studied rodent model of cGN, which is induced by the injection of sheep antibodies directed against the GBM of mice. This leads to the deposition of heterologous IgGs on the murine GBM as a planted antigen and induces innate and adaptive immune responses [34]. During the heterologous disease phase, which is characterized by cellular responses against the sheep IgGs, immune cells are recruited to the glomeruli and tubulointerstitium and induce glomerular damage and inflammation [35,36,37]. In the subsequent autologous phase of the disease, host antibodies develop against the heterologous IgGs on the GBM. Accelerated NTN is achieved by immunization in the presence of adjuvants before disease induction leading to a direct autologous reaction [38,39]. Apart from crescent formation, other pathological findings occur during NTN, including glomerular fibrin deposition and sclerosis, tubulointerstitial fibrosis as well as intracapillary or mesangial hypercellularity [10,40].

## 4. Pathogenic CD4^+^ T-Cell Responses in Immune-Mediated GN

Studies concerning athymic nude mice treated with human renal GBM provided the first evidence for a functional role of T cells in the pathogenesis of cGN. The authors showed that in the absence of T cells, autologous anti-GBM antibodies and glomerular injury did not develop [40]. Glomerular infiltration of T cells was first demonstrated in accelerated NTN induced in rats, in which the suppression of T-cell function by cyclosporine prevented glomerular injury [41]. The importance of T cells for the disease pathology of LN was described in MRL-*lpr* mice, which developed a less severe renal disease after T-cell depletion [42]. Later, the glomerular accumulation of CD4^+^ T cells was shown in accelerated NTN, and the treatment of rats with an anti-CD4 antibody prevented glomerular CD4^+^ T-cell infiltration resulting in reduced proteinuria and crescent formation [43]. This finding was confirmed in CD4^−/−^ mice, which did not develop proteinuria and showed decreased crescent formation compared to wild-type (WT) mice [44], demonstrating that glomerular injury depends on the CD4^+^ T cells in cGN. A critical role of CD4^+^ T cells in disease pathology has also been described in anti-MPO GN. Here, the depletion of CD4^+^ T cells reduced renal immune cell infiltration and attenuated cGN [45,46].

### 4.1. Th1-Cell Response

In NTN, the splenocytes of nephritic C57BL/6 mice were shown to produce high amounts of the Th1 effector cytokine interferon (IFN)γ [47]. Since the administration of an anti-IFNγ antibody [48] as well as lack of IFNγ in IFNγ^−/−^ mice [47,48] reduced crescent formation, this indicates a crucial role of Th1 cells in the development of glomerular injury. Moreover, it was also shown that intrinsic renal cells, such as tubular cells, contribute to the IFNγ response in NTN [49]. There is a substantial body of evidence linking the development of LN to Th1 cytokines. The inhibition of IFNγ signaling in NZB/NZW mice increased survival and decreased GN, while IFNγ treatment worsened disease [50]. Similar findings have been described for MRL-*lpr* mice [51,52,53], where IFNγ was shown to induce apoptosis in tubular epithelial cells [53]. Since pristane-treated IFNγ^−/−^ mice did not develop LN [54], these findings strongly indicate a pathogenic function of the Th1 response in LN. Furthermore, in anti-MPO GN, attenuation of the Th1 response by neutralization of IFNγ resulted in less severe cGN [55]. A strong polarization of Th1 cells has further been observed in IgAN and was correlated with the development of early renal injury in ddY mice [56]. Furthermore, the presence of autoreactive Th1 cells has been described during the progression from mild to severe cGN in EAG, and a lack of IFNγR reduced crescent formation and attenuated tubulointerstitial damage [57,58]. In contrast to this study, IFNγ^−/−^ mice showed an increased number of intraglomerular leukocytes and, despite a decreased autoantibody response, developed more severe EAG [59].

The importance of the Th1 response in NTN was underlined in experiments with either neutralization of the IFNγ-inducing cytokine IL-12 through the application of a monoclonal antibody directed against the p40 subunit of IL-12 or by a treatment with recombinant IL-12. The neutralization of IL-12 in C57BL/6 mice with accelerated NTN attenuated crescent formation and glomerular infiltration of CD4^+^ T cells, whereas application of IL-12 to mice with non-crescentic GN strengthened the Th1 response and induced severe cGN [60]. IL-12p40^−/−^ mice also showed a significant reduction in crescent formation and proteinuria [61], further highlighting the role of IL-12 in Th1 cell-mediated kidney injury. Mesangial cells and proximal tubular epithelial cells (PTECs) have been identified as a kidney-intrinsic source of IL-12 in accelerated NTN [62]. Since an IL-12 defect resulting in a high abundance of Th2 cytokines has been suggested to drive murine [63] and human LN [64], experiments with IL-12p35^−/−^ mice were performed to further assess the role of IL-12/IFNγ in disease pathology. In pristane-induced LN, the cytokine balance skewed towards a Th2 response in IL-12p35^−/−^ mice, which showed autoantibody production and renal immune complex deposition. However, pristane-treated IL-12p35^−/−^ mice did not develop proteinuria or renal damage [65], demonstrating the importance of the Th1 response for LN pathology.

The IL-12-induced immune response in NTN was shown to be enhanced by the application of IL-18, another Th1-prone cytokine [66]. In WT mice, the application of recombinant IL-18 augmented glomerular injury and immune cell accumulation. Since the treatment of IL-12p40^−/−^ mice with IL-18 restored crescent formation and glomerular CD4^+^ T-cell infiltration [61], this suggests that IL-18 acts synergistically but also independently of IL-12 to strengthen disease pathology of cGN. IL-18 has also been implicated in the pathogenesis of LN. MRL-*lpr* mice showed elevated levels of IL-18, and the treatment with recombinant IL-18 accelerated GN [67]. Moreover, survival and proteinuria were improved in MRL-*lpr* mice that lacked the expression of IL-18Rα or IL-18 [68,69], indicating a pathogenic role of IL-18 in LN.

The recruitment of CD4^+^ T cells into the kidney was shown to be mediated by IFNγ-inducible chemokines. During NTN, the renal expression of the chemokines CXCL9, CXCL10, and CXCL11 was induced, which are all ligands of the chemokine receptor CXCR3. Since CXCR3^−/−^ mice developed less severe NTN with reduced renal T-cell infiltration and IFNγ production [70], this indicates that CXCR3 expressed on T cells is required for their trafficking into the inflamed kidney, which expresses the corresponding chemokines after the release of IFNγ. The findings from the CXCR3^−/−^ mice were confirmed by another study revealing the importance of CXCL9, but not CXCL10, for CXCR3-mediated renal T-cells and macrophage infiltration during NTN [71]. The same group provided evidence for the renal expression of CXCL9 and CXCL10 in MRL-*lpr* mice, which showed an increased frequency of CXCR3-expressing T cells and macrophages in the inflamed kidney. However, the lack of CXCL10 in MRL-*lpr* mice did neither affect leukocyte infiltration nor the severity of LN [71], further demonstrating that CXCL10 is not central to the disease pathology of cGN. The expression of other chemokines such as CCL3, CCL4, and CCL5 was also upregulated in NTN, which induced the renal recruitment of T cells but also monocytes via the chemokine receptors CCR5 and CCR1 [72,73].

Initially, macrophages have been described as inducing glomerular injury and subsequent proteinuria in NTN [74,75,76]. Later studies found that the glomerular infiltration of T cells preceded the glomerular accumulation of inflammatory macrophages in accelerated NTN [41]. T cells from nephritic rats were shown to produce the macrophage migration inhibitory factor (MIF). Since glomerular T-cell infiltration and MIF production preceded macrophage influx, it was concluded that T cell-derived MIF induces macrophage localization within glomeruli during NTN [77]. Moreover, a lack of CD4^+^ T cells in nephritic rats and mice [42,43] as well as the inhibition of the Th1 response by blocking IFNγ [47,48], IL-12 [60,61], or IL-18 [61] resulted in reduced glomerular macrophage accumulation, demonstrating that Th1 cells regulate renal macrophage infiltration and thus, kidney injury in cGN. IFNγ produced by Th1 and renal intrinsic cells was shown to induce polarization of pro-inflammatory M1 macrophages [78,79], that in turn released IL-1β [80], thereby inducing the expression of another inflammatory cytokine, tumor necrosis factor (TNF)α. Although TNFα is also derived from T cells and activated macrophages, renal intrinsic cells, such as mesangial cells, glomerular cells, and tubular epithelial cells, have been described as a major source of this cytokine in NTN [81]. Macrophages were also shown to produce matrix metalloproteinase (MMP)-12 in glomeruli with crescent formations. Since treatment with an anti-MMP12 antibody reduced macrophage infiltration, crescent formation, and proteinuria, this indicates that MMP-12 is a macrophage-derived pathogenic factor in cGN [82]. The deposition of fibrin and the production of reactive oxygen species and iNOS have been described as further mechanisms by which macrophages contribute to glomerular damage [11].

**Figure 2:**
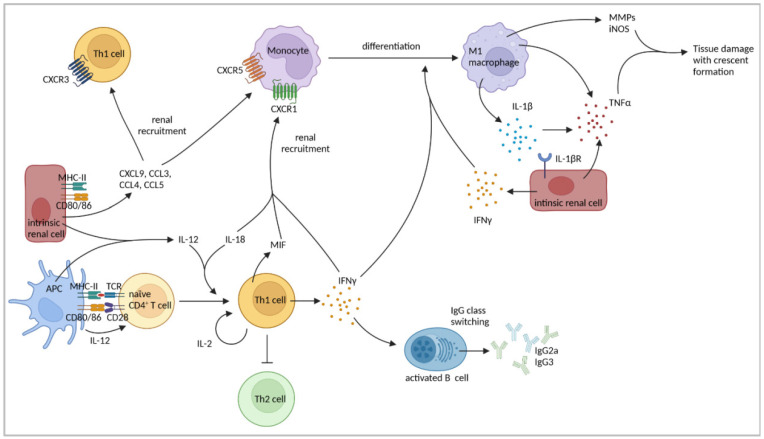
Th1 cell-mediated immune responses in cGN. Th1 cells and monocytes are recruited to the kidney via chemokines expressed by intrinsic renal cells. Professional and non-professional APCs express MHC-II and co-stimulatory molecules leading to antigen presentation and subsequent CD4^+^ T-cell activation. APC-derived IL-12 and IL-18 induce and reinforce Th1 cells resulting in enhanced expression of IFNγ and IL-2. IFNγ, produced by Th1 cells but also intrinsic cells, drives renal recruitment of macrophages and promotes differentiation of monocytes into inflammatory M1 macrophages. Macrophages produce IL-1β, thereby inducing TNFα expression by intrinsic cells and further mediating tissue damage and crescent formation through expression of matrix metalloproteinases (MMPs) and iNOS. Th1 cell-derived cytokines facilitate IgG class switching in B cells and inhibit Th2 responses. Figure has been created with BioRender.com (accessed on 23 March 2022).

Inflammation-induced renal accumulation of CD4^+^ T cells and macrophages was found to depend on the expression of major histocompatibility complex class II (MHC-II). MHC-II^−/−^ mice and chimeric mice, which expressed MHC-II only on hematopoietic cells, showed less glomerular immune cell infiltration and did not develop crescents, indicating an important role for MHC-II on renal intrinsic cells in immune cell recruitment during NTN [81]. Moreover, the expression of the co-stimulatory molecules CD80 and CD86 was detected in glomerular macrophages, endothelial cells, mesangial cells [83], and proximal tubules [84] in mice after NTN induction. The blockage of CD80/CD86 reduced disease severity and intraglomerular CD4^+^ T-cell and macrophage accumulation [83]. Thus, MHC-II, important for antigen presentation to CD4^+^ T cells, and CD80/CD86, which induce the co-stimulatory signal during T-cell activation, are required for the development of the Th1 response and subsequent NTN. A summary of the findings regarding the role of Th1 cells in cGN is presented in Figure 2 and Table 1.

### 4.2. Th2-Cell Response

NTN induction in Th2-prone BALB/c mice has been shown to result in a different pattern of GN than in Th1-prone C57BL/6 mice. BALB/c mice developed a proliferative, non-crescentic GN with a glomerular accumulation of neutrophils but with only a low infiltration of CD4^+^ T cells and macrophages [40]. Thus, while a pronounced Th1 response results in glomerular injury through recruitment and activation of CD4^+^ T cells and macrophages, the Th2 response induces glomerular neutrophil infiltration. Neutrophils have recently been described to be key pathogenic immune cells in GN, acting through different effector mechanisms, such as the production of reactive oxygen species, degranulation of serine proteases, or the formation of neutrophil extracellular traps [98]. In contrast to C57BL/6 mice, a strong linear deposition of mouse IgG has been detected in the glomerular capillary loops of nephritic BALB/c mice [40]. This finding confirmed earlier results demonstrating that Th2 cells promote humoral immune responses with antibody formation and class switching [99,100]. Splenocytes from nephritic BALB/c mice were shown to produce high amounts of IL-4, while IFNγ was less expressed [47], demonstrating the predominance of the Th2 response in this mouse strain. Although BALB/c mice did not develop crescents and fibrin deposits as pronounced as C57BL/6 mice, they also showed glomerular damage characterized by thickened capillary walls, expansion of the mesangium, and glomerular hypercellularity [47].

The application of the Th2 cytokine IL-4 to C57BL/6 mice attenuated crescent formation, proteinuria, and glomerular immune cell accumulation and led to reduced serum levels of the Th1 IgG subclasses IgG2a and IgG3 in NTN [101]. In contrast, the treatment of BALB/c mice with Th1 cytokine IL-12 exacerbated disease towards cGN with elevated proteinuria and glomerular fibrin deposition as well as CD4^+^ T-cell and macrophage accumulation. Moreover, elevated serum levels of IgG2a and reduced levels of the Th2 subclass IgG1 have been observed [60]. These findings indicate a mutual interference of Th1 and Th2 responses in cGN, which affects the outcome of the disease, and further highlight the importance of IL-12-induced Th1 responses for the development of crescents and glomerular macrophage accumulation. However, a lack of IL-4 in nephritic BALB/c mice did not lead to crescent formation [60], suggesting that other factors also dampen Th1 responses in BALB/c mice.

**Figure 3:**
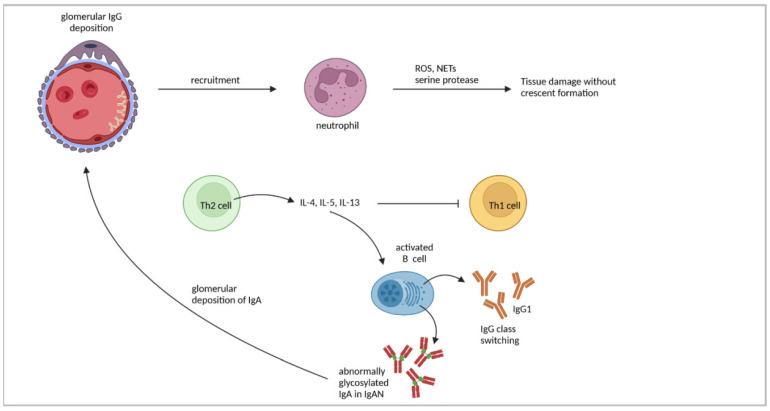
Th2 responses in immune-mediated GN. Glomerular immune complex deposition is a hallmark of Th2 cell-driven GN, leading to glomerular infiltration of neutrophils, which mediate tissue damage through production of reactive oxygen species (ROS), degranulation of serine proteases, and formation of neutrophil extracellular traps (NETs). Th2 cell-derived cytokines promote IgG class switching towards IgG1 in B cells, facilitate production of aberrantly glycosylated IgA, a characteristic of IgAN, and inhibit Th1 responses. Figure has been created with BioRender.com (accessed on 23 March 2022).

An imbalance of the Th1/Th2 cytokines has been described in IgAN, which might play a role in the development and progression of the disease [102]. A T cell-specific lack of Smad4 expression in *Smad4^co/co;Lck-cre^* mice led to an overproduction of Th2 cytokines and increased serum levels of IgA, which resulted in glomerular IgA deposition, aberrantly glycosylated IgA, and proteinuria [103], all features of human IgAN. Furthermore, ddY mice showed a strong Th1 response in the early stage of disease, while Th2 polarization was mainly observed in mice with quiescent disease [56]. Moreover, B cells produced higher levels of aberrantly glycosylated IgA in response to IL-4 and IL-5 in vitro [104,105], suggesting that a skewed Th2 response contributes to elevated levels of abnormally glycosylated IgA as observed in patients with IgAN [105,106]. The findings concerning Th2-cell responses are summarized in Figure 3 and Table 2.

### 4.3. Th17-Cell Response

The presence of renal Th17 cells has been described in NTN [109]. In order to determine the functional role of Th17 cells in the pathogenesis of cGN, NTN was induced in IL-23p19^−/−^ mice, which have reduced Th17 cell numbers. Compared to nephritic WT mice, IL-23p19^−/−^ mice showed a decreased frequency of renal Th17 cells, whereas the frequency of Th1 cells was unaltered. Moreover, IL-23p19^−/−^ mice developed less severe NTN with reduced crescent formation, proteinuria, and tubulointerstitial injury, as well as decreased glomerular immune cell infiltration [110]. The same phenotype has been described in nephritic IL-17A^−/−^ mice [110], indicating that in addition to the Th1 response, also Th17 cells and their effector cytokine IL-17A are pathogenic in NTN and contribute to the crescent formation and proteinuria.

The pathogenic potential of Th17 cells was also assessed in the NTN model. Here, the transfer of in vitro polarized Th17 cells to recombination activating gene 1 (Rag1)^−/−^ mice one day before NTN induction resulted in an enhanced renal infiltration of neutrophils and aggravated crescent formation and tubulointerstitial injury [111]. Since Rag1^−/−^ mice lack T and B cells, this model allows the analysis of in vitro polarized and subsequently transferred Th cells without any effects from endogenous T cells. In addition, RORγt, the key transcription factor in Th17-cell development, has been shown to promote cGN. Accelerated NTN was attenuated in RORγt^−/−^ mice, and the transfer of CD4^+^ T cells from RORγt^−/−^ mice into Rag1^−/−^ mice led to reduced crescent formation and necrosis, whereas CD4^+^ T cells from WT mice induced severe cGN [109].

The disease-driving potential of a Th17-cell response has also been shown in two models of autoimmune GN. In EAG, the progression of mild GN with less glomerular damage to severe cGN was accompanied by the renal infiltration of Th17 cells accumulating in the tubulointerstitium [57]. In addition, the presence of autoreactive renal Th17 cells was described in EAG. IL-17A^−/−^ and IL-23p19^−/−^ mice developed proteinuria and IgG deposition; however, they showed fewer glomerular crescents and reduced tubulointerstitial damage [58]. In another study, IL-23p19^−/−^ mice exhibited lower autoantigen levels, reduced IL-17A production and glomerular IgG deposition, as well as less severe EAG [29]. The immunization of WT mice with MPO was found to induce systemic IL-17A production, and after the injection of anti-GBM antibodies, the glomerular deposition of MPO was achieved by infiltrating neutrophils leading to anti-MPO GN. In contrast to WT mice, IL-17A^−/−^ mice were protected and did not develop anti-MPO GN, which was attributed to the reduced neutrophil infiltration and subsequent decrease in the deposition of glomerular MPO [112].

An elevated number of renal Th17 cells and an enhanced expression of IL-17A have also been described in ddY mice developing IgAN [113]. In MRL-*lpr* mice, T cells expressed high levels of IL-17A and IL-23R, which increased along with the progression of LN [114]. The blockage of the Th17 cell-inducing cytokine IL-23 in MRL-*lpr* mice diminished IL-17A expression by splenocytes and proteinuria, while autoantibody production was not affected [115]. In contrast, the transfer of lymphocytes from lupus-prone mice, which were pre-treated with IL-23, into Rag1^−/−^mice, lacking T cells and B cells, induced nephritis [114], indicating that the IL-23/IL-17A axis contributes to the development of LN. Moreover, lack of TNF receptor (TNFR)1/TNFR2 expression in NZB/NZW mice accelerated LN, which was correlated with an elevated number of Th17 cells [116].

The pathogenic function of Th17 cells in comparison to Th1 cells was analyzed in Rag1^−/−^ mice, to which the model antigen ovalbumin (OVA) was planted on the GBM by injection of an OVA-conjugated non-nephritogenic antibody against α3(IV) collagen [87]. Following the transfer of OVA-specific Th1 or Th17 cells, the mice developed GN without an additional application of nephrotoxic serum. While the glomerular and interstitial accumulation of CD4^+^ T cells and macrophages was similar after the transfer of Th1 or Th17 cells, elevated numbers of neutrophils within glomeruli and the tubulointerstitium were only detected after Th17 transfer. Accordingly, the expression of the neutrophil-attracting chemokine CXCL1 was enhanced in the kidneys of mice receiving Th17 cells, whereas transfer of Th1 cells resulted in elevated expression of CCL2 and CCL5. While transferred Th17 and Th1 cells induced proliferative GN, only Th1 but not Th17 cells induced a crescent formation. Both cell populations induced sustained proteinuria, but the mice that received Th17 cells developed proteinuria much earlier, which was associated with a higher number of neutrophils within glomeruli [87]. Although there is evidence that Th17 cells can convert into Th1 cells in autoimmune disease [117], the in vitro polarized Th17 cells still expressed IL-17A on day 21 after transfer [87], indicating that their phenotype persists in nephritic kidneys. This was further underlined in NTN and pristane-induced LN, where Th17 cells did not start to express IFNγ during the course of the disease [111]. These findings demonstrated that both Th17 and Th1 cells are capable of inducing a proliferative GN. However, the kinetic and outcome of injury, as well as the predominance of cellular and soluble effectors, are different. While Th17 cells induce early glomerular damage, probably through CXCL1-mediated neutrophil recruitment, Th1 cells induce glomerular injury and crescent formation at later time points, which is associated with renal recruitment of macrophages, possibly by CCL2 and CCL5.

The time course of Th17 cell-induced kidney damage was further analyzed by NTN induction in IL-17A^−/−^ mice. On day six of the disease, IL-17A^−/−^ showed less crescent formation and a diminished accumulation of CD4^+^ T cells, macrophages, and neutrophils than the nephritic WT mice. On day 14, this protection from renal injury could not be detected anymore, and on day 21, the IL-17A^−/−^ mice depicted more severe cGN than WT mice. At earlier time points of the disease, IL-17A deficiency led to an increased expression of IL-4 by splenocytes, whereas IFNγ production was not altered. Later, systemic IFNγ expression was much more elevated than IL-4 production, indicating that the Th1/Th2 balance was shifted towards a Th1 response in the absence of IL-17A. In addition, more severe cGN was also determined in IL-23p19^−/−^ mice on day 21 of the disease [86]. These findings reveal that IL-17A exacerbates early glomerular injury but attenuates established cGN by suppressing the Th1 response. This was further underlined in nephritic IL-12p35^−/−^ mice, which showed less severe NTN than WT mice on day 21 of the disease. In these animals, systemic IFNγ production was lower than in WT mice, whereas IL-17A secretion was increased [86]. A similar biphasic Th-cell response was observed in anti-MPO GN. An early Th17 and a late Th1 response were demonstrated in IL-23p19^−/−^ and IL-12p35^−/−^ mice [91]. In a therapeutic approach, treatment with an anti-IL-23p19 antibody blocked early immune responses and glomerular injury without affecting the late Th1 response, whereas an anti-IL-12p35 antibody did not influence early autoimmunity but attenuated Th1 cell-mediated immunity. Interestingly, the administration of a monoclonal antibody directed against p40, which represents the common subunit of IL-23 and IL-12, was effective in both phases of anti-MPO GN [91], providing experimental evidence for a treatment option in autoimmune GN.

IL-17A has been shown to induce the expression of chemokines CCL2, CCL3, and CCL20 in mouse mesangial cells in vitro [110], among which CCL20 has been implicated in the recruitment of Th17 cells expressing the chemokine receptor CCR6 [118]. In a later study, the upregulation of CCL20 expression in NTN was determined. This was associated with the renal infiltration of Th17 cells, which was abrogated in CCR6^−/−^ mice [38], demonstrating the importance of CCL20/CCR6 for renal Th17 cell accumulation in cGN. An elevated renal expression of CCL20 has also been shown in ddY mice, and neutralization of CCL20 decreased the Th17-cell number, IL-17A levels, and renal damage in IgAN [109].

Th17 cells are abundant in the intestine, where they are induced by commensal bacteria and provide protection against invading pathogens. In autoimmune disease, the migration of intestinal Th17 cells to affected organs, e.g., the central nervous system, has been described [119]. A potential translocation of gut-derived Th17 cells to the nephritic kidney has been analyzed in mice, which ubiquitously express Kaede, a photoconvertible fluorescent protein related to a green fluorescent protein that irreversibly switches its emission light from green to red after exposure to light with a distinct wavelength. In NTN, the intestine of Kaede mice was exposed to light before analysis on day seven of the disease. In the kidneys of nephritic Kaede mice, red fluorescent Th17 cells were identified, demonstrating their inflammation-induced migration from the gut to the kidney. In contrast, Th1 cells were not derived from the intestine in nephritic kidneys. The study also revealed a sphingosine-1 phosphate receptor 1-mediated emigration of Th17 cells from the gut and a CCL20/CCR6-induced infiltration of Th17 cells into the kidney [120]. Interference with the microbiome, either by using germ-free mice or by antibiotic treatment, strongly reduced the intestinal Th17-cell number and subsequent renal Th17-cell recruitment and ameliorated the disease pathology of NTN. On the contrary, the infection of mice with *Citrobacter rodentium* resulted in aggravated NTN with an enhanced renal Th17 response [120], indicating an impact of the microbiome on disease progression of cGN by regulating the Th17 response.

IL-17F, another cytokine produced by Th17 cells, has also been shown to mediate tissue injury during cGN. IL-17F has been implicated in the induction of the neutrophil-attracting chemokines CXCL1 and CXCL5. Accordingly, the number of renal infiltrating neutrophils was reduced in nephritic IL-17F^−/−^ mice that developed less severe NTN [121]. In addition, the expression of IL-17C was determined in renal intrinsic cells but not lymphocytes in NTN. The IL-17C receptor IL-17RE was expressed by Th17 cells, and a lack of either IL-17C or IL-17RE ameliorated the course of the disease [122]. These findings indicate that the IL-17C/IL-17RE axis promotes Th17 responses in cGN. Moreover, the expression of the IL-17 receptor A/C complex by Th17 cells and the activation of the IL-17R signaling pathway have been described in NTN. A lack of IL-17RC expression in Th17 cells led to an elevated cytokine response and aggravated the disease pathology [123], indicating a regulatory effect of IL-17RC signaling on Th17 cell function in cGN.

**Figure 4:**
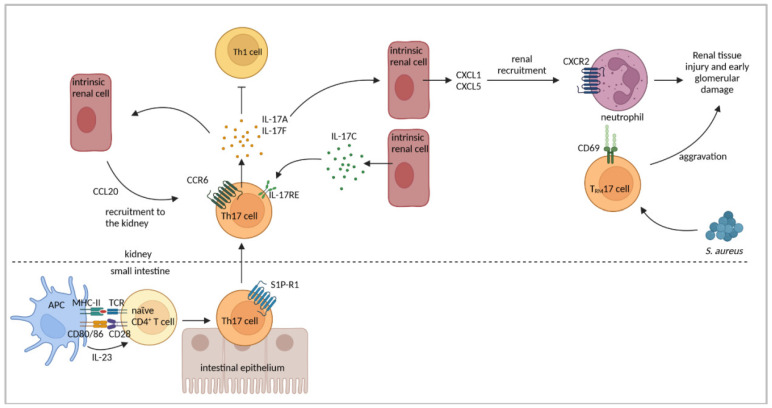
Th17 cell-mediated immune responses in cGN. Th17 cells are polarized in the intestine, from which they emigrate in a sphingosine-1 phosphate receptor (S1P-R)1-dependent manner. Renal intrinsic cells produce the chemokine CCL20 to recruit CCR6^+^ Th17 cells into the kidney. Th17 cell-derived cytokines induce a positive feedback loop for Th17-cell infiltration by induction of CCL20 in intrinsic cells. IL-17A and IL-17F further promote production of neutrophil-attracting chemokines leading to accumulation of tissue-damaging neutrophils in the kidney. Renal intrinsic cells also express IL-17C, which promotes Th17 responses through IL-17RE. Th1 responses are inhibited by Th17 cells. Renal infection with *Staphylococcus aureus* (*S. aureus*) induces T_RM_17 cells, which persist in the kidney after pathogen clearance, and aggravate renal pathology in cGN. Figure has been created with BioRender.com (accessed on 23 March 2022).

An early Th17 response followed by a Th1 response has also been observed in other immune-mediated diseases such as experimental autoimmune encephalomyelitis [124,125] and type 1 diabetes mellitus [117,126]. Interestingly, these studies indicate that Th17 cells convert to Th1 cells giving proof of Th17-cell plasticity [127]. Based on these findings, the plasticity of Th17 cells has been studied in cGN. As mentioned above, Th17 cells showed a stable phenotype overtime after the transfer into nephritic *Rag1*^−/−^ mice [87]. However, Th17 cells were induced to produce the anti-inflammatory cytokine IL-10 after the treatment of nephritic mice with an anti-CD3 antibody [111], an effect which has already been shown for T cells in the intestine [128]. Th17 cells still expressed IL-17A after anti-CD3 antibody treatment, but renal damage was less severe [111]. Since the conversion of Th17 cells into IL-10-expressing regulatory Tr1 cells has also been show in NTN [129], this suggests a suppressive effect of Th17 cell-derived IL-10 on disease pathology. However, the data presented so far point away from a regulatory role of IL-10^+^ Th17 cells in cGN [129]. Figure 4 and Table 3 summarize the key findings regarding the role of Th17 cells in immune-mediated GN.

### 4.4. Tissue-Resident Memory CD4^+^ T Cells

A recently identified subset of T-cells are tissue-resident memory T (T_RM_) cells, which constitute the most abundant memory T-cell subset residing in tissue. T_RM_ cells participate in protection from infection and cancer, but probably promote autoimmune and inflammatory disease [137]. T_RM_ cells express typical markers of tissue residency, i.e., CD69, CD49a, and sometimes CD103, all of them mediating retention in tissues. T_RM_ cells further express transcription factors such as a homolog of Blimp-1 in T cells (Hobit) and Blimp-1, which repress the expression of proteins crucial for tissue egress [138].

Local infections and their role in the pathogenesis of autoimmune disease have been linked to the bystander activation and expansion of previously activated T cells at an inflamed site [139]. Increasing evidence argues that CD4^+^ T_RM_ cells play a role in this process of immune surveillance [137]. Recently, CD4^+^ T_RM_ cells have been identified in the kidneys of patients with ANCA-associated GN. Single-cell RNA-seq and CITE-seq analyses revealed a Th1 and Th17 cell signature of these cells. In addition, the number of CD4^+^ T_RM_ cells correlated with the impairment of kidney function in patients with ANCA-GN [92]. In an experimental mouse model, renal infection with *Staphylococcus aureus* (*S. aureus*) induced CD4^+^ T_RM_ cells bearing a Th17 signature (T_RM_17). These cells persisted when the infection was cleared. The induction of NTN following the elimination of the pathogen resulted in an aggravated renal pathology. Intriguingly, renal but not splenic T_RM_17 cells were sensitive to activation by Th17-polarizing cytokines in the absence of TCR signaling, and neutralization of these cytokines during NTN reduced crescent formation in *S. aureus* pre-infected animals [92]. This study provides evidence for the role of pathogen-induced T_RM_17 in the aggravation of cGN.

### 4.5. CD4^+^ T-Cell Responses in GN Patients

In ANCA-associated GN, autoreactive CD4^+^ T cells recognizing MPO have been identified as critical mediators of autoimmunity and renal injury [45,46,140,141]. Patient-derived blood lymphocytes were found to produce IFNγ, suggesting that the Th1 response drives autoimmunity in ANCA-associated GN [93]. However, elevated numbers of blood Th17 cells and enhanced levels of Th17 cell-associated cytokines, which correlated with disease activity, have also been described [134,135]. Moreover, a high frequency of Th17 cells was determined in the kidneys of patients with ANCA-associated GN [120], indicating that both Th1 and Th17 responses contribute to disease pathology.

In IgAN, higher proportions of circulating Th2 and Th17 cells but lower frequencies of Th1 cells have been determined [94,95,96]. An elevated numbers of Th17 cells in the blood and increased serum levels of IL-17A have been shown in patients with IgAN [95,96]. In addition, IL-17A expression was found at renal tubule sites in more than half of the analyzed patients with IgAN, and particularly these patients had decreased renal function and increased proteinuria and tubulointerstitial damage [94], indicating a detrimental role of Th17 cells in IgAN. Genetic studies showed an association between IFNγ polymorphism and higher susceptibility to the development of IgAN. The polymorphism was determined in the binding site for transcription factor NF-κB, which was associated with decreased NF-κB binding affinity and decreased IFNγ production in response to stimulation in vitro [97], suggesting a protective role of IFNγ in IgAN. One characteristic of IgAN is the appearance of aberrantly glycosylated IgA1. Similar to studies in mice, the Th2 cell-associated cytokine IL-4 was found to promote IgA1 production in human B cells and promoted the higher secretion of aberrantly glycosylated IgA1 [107,108]. Such induced alterations in the glycosylation of IgA1 have also been described for IL-17A [136], indicating that Th2 and Th17 cells support IC-mediated GN and renal tissue damage.

An imbalance of Th subsets has been suggested to contribute to the pathogenesis of SLE. Elevated serum levels of IL-12 [88,89,90] IL-17A [88,90,130] and IL-23 [89,131] were determined in SLE patients, indicating Th1 and Th17 cell-mediated immunity. Glomerular expression of IL-17A and IFNγ was detected in patients with LN, who also showed elevated ratios of Th1/Th2 and Th17/Th2 cytokines [90]. Th17 cells have been detected within glomeruli and in the tubulointerstitium of patients with LN and correlated with disease parameters, including blood urea nitrogen and serum creatinine levels and the SLE disease activity index score [130]. Double negative (DN) CD3^+^ CD4^-^ CD8^-^ T cells have also been identified as producers of IL-17A and IFNγ in SLE, and particularly IL-17A^+^ DN T cells were found in the kidneys of patients with LN [133], suggesting that they contribute to kidney damage in SLE.

## 5. Pathogenic CD8^+^ T-Cell Responses in cGN

### 5.1. Effector CD8^+^ T Cells

In patients with ANCA-associated GN and LN, CD8^+^ T cells have been shown to infiltrate the kidney in similar or even enhanced numbers compared to CD4^+^ T cells [4,5,6,7,142]. Periglomerular CD8^+^ T-cell accumulation correlated with crescents and BC rupture as well as with the disease activity score and high serum creatinine levels in LN [7], suggesting a pathogenic role of CD8^+^ T cells in cGN. Moreover, CD8^+^ T cells from patients with LN were clonally expanded and persisted for years in repeated renal biopsies during flares [6], probably being responsible for disease progression. Mapping of the immune cell landscape in kidneys of patients with LN and healthy controls by scRNA sequencing identified two clusters of cytotoxic CD8^+^ T cells expressing high levels of either granzyme B (GzmB) and perforin or GzmK in the diseased kidneys [143]. In addition, transcriptional profiling of blood CD8^+^ T cells from AAV patients provided evidence for a correlation of an activated, non-exhausted phenotype of CD8^+^ T cells with a poor prognosis of the disease [144,145].

Early functional studies in rats demonstrated a role of CD8^+^ T cells in NTN since, after antibody-mediated CD8^+^ T-cell depletion, reduced crescent formation, proteinuria, and glomerular macrophage infiltration have been observed [146]. In a follow-up study in rats, the depletion of CD8^+^ T cells during EAG was shown to prevent renal disease and was even identified as a treatment option for already-existing disease [147]. In contrast to these studies pointing to a pathogenic function of CD8^+^ T cells in cGN, CD8^−/−^ mice were not protected from NTN and instead developed accelerated glomerular disease. The authors suggested that the reason for disease aggravation in CD8^−/−^ mice could be related to an expansion of CD4^+^ T cells into niches, which are otherwise occupied by CD8^+^ T cells in WT mice. This was evidenced by increased autologous antibody deposition in glomeruli and enhanced glomerular macrophage recruitment, indicating an elevated CD4^+^ Th response in the absence of CD8^+^ T cells [6]. In addition, the mice deficient in the transporters associated with antigen processing, which have a strongly reduced ability to present antigen by MHC-I and, therefore, harbor a reduced number of CD8^+^ T cells, were not protected from NTN [148]. In the same study, the authors showed that antibody-induced CD8^+^ T-cell depletion failed to prevent the crescent formation in mice. However, it reduced the proteinuria and glomerular recruitment of CD4^+^ T cells and macrophages in NTN [148].

These observations in rodent models, providing inconsistent results utilizing heterologous anti-GBM antibodies to induce cGN, were later extended by studies using the murine model of autoimmune anti-MPO GN. Here, immunization with either MPO or the immunodominant MPO peptide elicited the production of anti-MPO antibodies and induced the expansion of autoreactive, MPO-specific CD4^+^ T cells, which mediated cGN. However, the depletion of CD8^+^ T cells following MPO immunization attenuated proteinuria and segmental necrosis as well as CD4^+^ T-cell and macrophage recruitment into glomeruli in anti-MPO GN. Moreover, the transfer of MPO-specific CD8^+^ T cells into Rag1^−/−^ mice prior to disease induction induced renal disease, while co-transfer of MPO-specific CD8^+^ T cells and CD4^+^ T cells worsened anti-MPO GN [149]. Thus, renal autoantigen expression may result in the recruitment of autoantigen-specific CD8^+^ T cells that aggravate cGN. Whether the autoantigen is presented by local DCs or probably by non-professional antigen-presenting cells (APCs) in the tubular system or glomeruli has not yet been investigated. The findings were supported by another study using transgenic mice, in which podocytes specifically expressed the model antigen-enhanced green fluorescent protein (EGFP). The transfer of EGFP-specific CD8^+^ T cells together with the induction of NTN induced severe cGN. Interestingly, CD8^+^ T cells only infiltrated into the glomeruli displaying a disrupted BC and induced the cell death of podocytes [15]. Hence, CD8^+^ T cells may be responsible for disease progression of autoimmune cGN if the BC is destroyed during the inflammatory process and an autoantigen is presented by podocytes. Indeed, it has been shown that podocytes can function as non-professional APCs [150]. However, the transfer of antigen-specific CD8^+^ T cells alone did not mediate cGN and required injection of anti-GBM antibodies to induce NTN. This could imply that DCs, which are activated during inflammation, encounter neo-antigen from dying podocytes and cross-present it to CD8^+^ T cells, which in turn kill podocytes by a yet undefined mechanism [14].

A study described the generation of albumin-derived peptides that probably function as autoantigens in proteinuric nephropathy, where excess filtration of albumin is due to a breakdown of the glomerular filtration barrier. Exposure of rat PTECs to albumin resulted in its proteolytic cleavage and release of protein fragments that were further processed by DCs in vitro. The incubation of bone marrow-derived DCs with a PTEC-derived peptide enabled them to activate syngeneic CD8^+^ T cells, demonstrating the antigenic nature of the presented peptide. The inhibition of the proteasome prevented DC-mediated CD8^+^ T-cell activation, indicating that DCs process PTEC-derived protein fragments into smaller peptides, which are cross-presented to CD8^+^ T cells via MHC-I, leading to their activation [151]. Endocytosis of albumin by PTECs further induced the activation of NF-κB and MAP kinases and, in turn, production of cytokines and chemokines by these cells [152], which might contribute to leukocyte recruitment in renal disease. In the model of five-sixths nephrectomy in rats, DCs infiltrated into renal parenchyma and then migrated to the renal draining lymph node. DCs isolated from renal lymph nodes, loaded with the PTEC-derived peptide, activated CD8^+^ T cells in vitro, which was abrogated after proteasome inhibition [151]. These findings reveal the generation of antigenic peptides from the self-protein albumin through a concerted action of PTECs and DCs, which might participate in the induction of autoimmune kidney disease. Since PTECs have been described as a population of non-professional APCs in the kidney [84], they might also participate in the process of autoantigen presentation to CD8^+^ T cells. It should be emphasized that in AAV-associated GN as well as in NTN, where albuminuria is common, most of the T cells are located in the periglomerular and tubulointerstitial space. Therefore, one could speculate that a similar mechanism of CD8^+^ T-cell activation by albumin-derived self-antigens might also contribute to cGN.

### 5.2. Tissue-Resident Memory CD8^+^ T Cells

Renal CD8^+^ T_RM_ cells have recently been identified in human and murine renal-cell carcinoma, and it has been suggested that they provide anti-tumor activity [153,154]. In the kidneys of patients with LN, one cluster of CD8^+^ T_RM_ cells has been described that highly expressed the T_RM_ markers Hobit, CD103, and CD49a but lacked the expression of KLF2, which is responsible for tissue egress. In contrast, the CD4^+^ T_RM_ cell cluster were not detected [143]. Exhausted CD8^+^ T cells, including CD8^+^ T_RM_ cells, as they have been detected in kidneys of lupus-prone mice [155] and in the peripheral blood of LN patients, were not present in the kidneys of nephritic patients [143]. These findings were substantiated by a follow-up study that also identified renal CD103^+^ CD8^+^ T_RM_ cells in patients with LN [156]. CD8^+^ T_RM_ cells were further detected in the kidneys of MRL-*lpr* mice, whereas they were rarely present in the blood and spleen of these mice. CD8^+^ T_RM_ cells expressed the classical T_RM_ markers, CD69 and CD103. They increased during the course of the disease, and their number correlated with the severity of LN. Moreover, renal CD8^+^ T_RM_ cells in MRL-*lpr* mice were not exhausted as they expressed GzmB and perforin as well as the inflammatory cytokines IFNγ and TNFα [156], suggesting that they might contribute to LN pathology.

**Figure 5:**
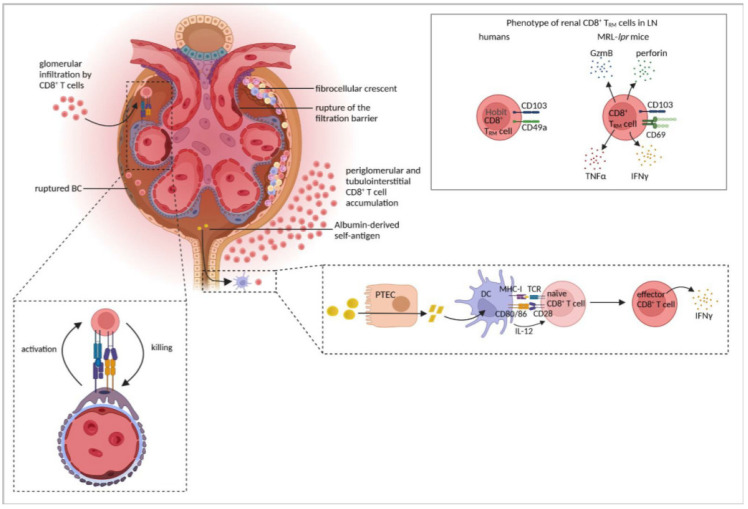
CD8^+^ T-cell responses in cGN. CD8^+^ T cells infiltrate the kidney and accumulate in the tubulointerstitium and periglomerular region. In severe cGN, breaches in the Bowman´s capsule lead to glomerular CD8^+^ T-cell infiltration, podocyte killing, and subsequent rupture of the filtration barrier. Antigen presentation by podocytes induces activation of infiltrating CD8^+^ T cells, which in turn kill the podocytes. Albumin might function as a self-antigen in proteinuric nephropathy, which is processed by proximal tubular epithelial cells (PTECs) and DCs into antigenic peptides, which induce CD8^+^ T-cell activation and production of IFNγ. Renal CD8^+^ T_RM_ have been described in lupus nephritis (LN). In humans, CD8^+^ T_RM_ are characterized by expression of the transcription factor Hobit and the tissue retention molecules CD49a and CD103. In mice, CD8^+^ T_RM_ express cytotoxic molecules and inflammatory cytokines and express CD69, another molecule associated with tissue residency. Figure has been created with BioRender.com (accessed on 23 March 2022).

So far, little is known about mechanisms leading to the activation of CD8^+^ T_RM_ cells in a given disease. Interestingly, auto-aggressive CD8^+^ T_RM_ cells that exert their effector functions independently of T-cell receptor (TCR) activation but that are dependent on the P2X-purine receptor 7-dependent upregulation of FasL have been recently described to mediate immunopathology in a mouse model of metabolic liver injury [157]. Hence, as it has been described for renal CD4^+^ T_RM_ cells, which were activated by inflammation-induced cytokines [92], also CD8^+^ T_RM_ cells might probably be activated independently of TCR signaling in cGN. Figure 5 and Table 4 summarize findings with respect to the role of CD8^+^ T cells in immune-mediated GN.

## 6. Conclusions and Therapeutic Considerations

Current therapies to achieve a remission in immune-mediated GN mainly rely on immunosuppressive drugs and steroids [16]. These drugs may have serious side effects and are rather unspecific for the individual GN, which can become refractory to the treatment. Therefore, pre-clinical and clinical research aims to unravel shared and distinct immunological mechanisms involved in the pathogenesis of individual GNs in order to identify targets for novel therapies, at least with the intention of lowering standard therapy. Compelling evidence from experimental studies in mouse models of ANCA-associated GN, Goodpasture’s disease, LN, and IgAN point to a major role of the Th17 response in the pathogenesis of cGN. Similarly, clinical data showing elevated numbers of Th17 cells and enhanced levels of Th17 cell-associated cytokines in serum or renal tissue have been widely documented, which correlated with disease activity in ANCA-associated GN, IgAN, and LN [88,89,90,94,95,96,120,130,131,132,134,135]. Therapies affecting the IL-17/IL-23 axis were proven to be effective for the treatment of plaque psoriasis, psoriatic arthritis, and ankylosing spondylitis [158]. Secukinumab, a human monoclonal antibody against IL-17A, is currently being evaluated in a phase III study for efficacy and safety in combination with standard therapy in patients with active LN [159]. The primary endpoint of this study will be the percentage of patients achieving a complete renal response (CRR). Interestingly, few case reports described renal protection due to secukinumab treatment for psoriasis in patients with co-existing refractory LN [160]. Brodalumab, a human monoclonal antibody against IL-17RA, which mediates the downstream signaling of IL-17A, IL-17F, IL-17A/F, IL-17C, and IL-17E, has been approved for the treatment of psoriasis [161]. Since, in addition to IL-17A, IL-17F and IL-17C, which have been shown to mediate experimental cGN [121,122], the treatment of cGN patients with brodalumab could represent a promising future therapeutic strategy.

Ustekinumab, a monoclonal antibody that recognizes the common p40 subunit of IL-12 and IL-23, has been approved for the treatment of psoriasis, psoriatic arthritis, and Crohn’s disease [158]. In a multicenter phase II study, ustekinumab has been evaluated for efficacy and safety in patients with active SLE. Ustekinumab was shown to improve the efficacy of standard therapy in addition to lowering the risk of new flares [162]. Up to now, there are no clinical reports for the use of ustekinumab in LN [163]. Again, pre-clinical and clinical data should encourage clinical studies with ustekinumab since, in addition to the Th17 response, Th1 cells are also involved in kidney injury in human and murine ANCA-associated GN and LN, and blockade of p40 has been shown to prevent experimental cGN [91].

Belimumab is the only biological agent that has been approved by the FDA for the treatment of LN so far. This monoclonal antibody is directed against the B cell-activating factor (BAAF), leading to impaired B-cell survival [164]. The antibody has been previously approved for the treatment of SLE and was proposed as a combination therapy together with the B cell-depleting anti-CD20 antibody, rituximab, where 67% of the patients with LN were complete renal responders [165,166].

In addition to the important role of T-cell immunity in ANCA-associated vasculitis and nephritis, the activation of the alternative complement pathway and the ensuing production of anaphylatoxins seem to be critically involved in the disease. A hypothetical molecular link between T-cell activation and the formation of anaphylatoxins, such as C3a and C5a, by activated neutrophils, may be related to Th17 cell-dependent neutrophil activation and its consecutive acceleration by the anaphylatoxins themselves [167]. Molecules that activate the alternative complement pathway, such as properdin, are produced by activated neutrophils, and the glomerular deposition of properdin associated with cellular crescents and proteinuria was detected in patients with ANCA-associated GN [160,168]. The identification of this pathway in the pathogenesis of ANCA-associated GN prompted the clinical development of avacopan, a C5a receptor antagonist [167].

Autoimmunity also results from dysfunctional regulatory T cells (Tregs) that are impaired in their capacity to suppress the effector function of activated T cells. The majority of Tregs represent a subset of CD4^+^ T cells expressing the forkhead box P3 transcription factor as well as high levels of the IL-2 high-affinity receptor IL-2Rα or CD25 [169]. Reduced Treg numbers have been positively linked with disease activity in SLE patients and in lupus-prone mice and were associated with an impaired capacity of T cells to produce IL-2 [170,171,172,173,174]. IL-2 has been implicated in the survival, suppressive function, and expansion of peripheral Tregs [175,176,177]. In addition, IL-2, by activation of STAT5, directly inhibits STAT3-mediated differentiation of Th17 cells [178]. These observations encouraged clinical studies using low-dose IL-2 as a therapy in SLE patients, which resulted in an expansion of peripheral Tregs expressing high levels of CD25, retained their suppressive capacity and reduced disease activity [172,179]. Another clinical study with low-dose IL-2 together with rapamycin in five refractory SLE patients reported relief from SLE symptoms assessed by the SLEDAI score along with the expansion of Tregs and reduction in Th17/Treg ratios [180]. Up to now, studies investigating the clinical efficacy and safety of low-dose IL-2 in LN have not been published.

Taken together, the overactivation of effector T-cell immunity is fundamental in the pathogenesis of immune-mediated GN. Novel insights into renal T-cell biology, particularly with respect to local immune responses in the kidney, would hopefully unravel novel targets for future therapies, such as molecules that affect expansion and effector functions of CD4^+^ T_RM_ and CD8^+^ T_RM_ cells on the one hand, and allow Treg function and stability on the other hand. Table 5 summarizes the immunomodulators approved or in clinical development for treatment of cGN.

## Figures and Tables

**Table 1:** Th1-cell responses in human and murine cGN.

Type of GN	Key Findings	Refs
NTN	IFNγ, secreted by Th1 cells, but also renal intrinsic cells, promoted crescent formation.	[47,48,49]
	Neutralization of the p40 subunit of IL-12 attenuated crescent formation and glomerular CD4^+^ T-cell infiltration.	[60]
	IL-12p40^−/−^ mice showed reduced crescent formation and proteinuria.	[61]
	Application of IL-12 in non-crescentic mice worsened disease towards cGN.	[60]
	Tubular epithelial cells and mesangial cells produced IL-12.	[62]
	IL-18 enhanced immune response induced by IL-12.	[66]
	Treatment of IL-12p40^−/−^ mice with IL-18 restored crescent formation.	[61]
	CXCR3^−/−^ mice developed less severe NTN with reduced IFNγ production and renal T-cell infiltration.	[70]
	CXCL9 induced CXCR3-mediated macrophage and T-cell recruitment.	[71]
	CCL3, CCL4, and CCL5 were upregulated, leading to renal monocyte and T-cell recruitment via CCR5 and CCR1.	[72,73]
	Macrophages induced glomerular injury.	[74,75,76]
	Glomerular macrophage accumulation was preceded by glomerular T-cell infiltration.	[41]
	T cell-derived MIF induced macrophage accumulation within glomeruli.	[77]
	Lack of CD4^+^ T cells resulted in reduced macrophage accumulation within glomeruli.	[42,43]
	Blocking of IFNγ, IL-12 or IL-18 diminished glomerular macrophage accumulation.	[47,48,60,61]
	IFNγ production by Th1 and renal intrinsic cells induced pro-inflammatory M1-macrophage polarization and production of IL-1β and TNFα by macrophages.	[78,79,80]
	Renal intrinsic cells were identified as major source of TNFα.	[85]
	MHC-II expression on renal intrinsic cells was important for T-cell and macrophage recruitment.	[81]
	Blockage of CD80/CD86 reduced intraglomerular accumulation of CD4^+^ T cells and macrophages.	[83]
	IL-12p35^−/−^ mice showed less severe NTN on day 21 of disease, but increased IL-17A expression.	[86]
OVA as GBM-fixed antigen in mice	Transfer of OVA-specific Th1 cells induced cGN, macrophage recruitment, and tissue injury at a later time point than after Th17-cell transfer. Renal CCL2 and CCL5 expression were elevated after Th1 cell transfer.	[87]
NZB/NZW mice	Treatment of mice with IFNγ promoted disease progression.	[50]
	An IL-12 defect resulted in more severe LN.	[63]
MRL-*lpr* mice	IFNγ induced apoptosis in tubular epithelial cells.	[53]
	Defect in IL-12 production by macrophages led to high levels of type 2 cytokines and may drive LN.	[63]
	IL-18 levels were elevated and IL-18 accelerated GN.	[67]
	Lack of IL-18Rα or IL-18 improved proteinuria and survival.	[68,69]
	CXCR3-mediated macrophage and T-cell recruitment were dependent on CXCL9.	[71]
Pristane-induced murine LN	IFNγ^−/−^ mice did not develop LN.	[54]
	IL-12p35^−/−^ mice did not develop glomerular damage and proteinuria.	[65]
Human LN	Reduced production of IL-12 and IFNγ resulted in higher levels of type 2 cytokines and may drive LN.	[64]
	IL-12 serum levels were elevated in SLE patients.	[88,89,90]
Murine anti-MPO GN	Neutralization of IFNγ led to less severe cGN.	[55]
	The Th1 response developed following an early Th17 response.	[91]
	Treatment of mice with anti-IL-12p35 antibody blocked late GN.	[91]
Human ANCA-GN	Identification of CD4^+^ T_RM_ cells that showed a Th1 and Th17 signature and correlated with renal failure.	[92]
	Patient-derived blood lymphocytes expressed IFNγ.	[93]
Human IgAN	Th1 polarization was observed in IgAN patients.	[56]
	Higher frequencies of blood Th2 and Th17 cells, but lower proportion of Th1 cells were shown.	[94,95,96]
	IFNγ polymorphism led to decreased NF-κB binding affinity and less IFNγ production associated with higher susceptibility to IgAN development.	[97]
ddY mice	Strong Th1 response developed in early disease.	[56]
	A Th1 polarization correlated with early renal injury.	[56]
Murine EAG	Autoreactive Th1 cells led to the progression from mild to severe cGN.	[57,58]
	IFNγ^−/−^ mice developed more severe cGN.	[59]

**Table 2:** Th2-cell responses in human and murine cGN.

Type of GN	Key Findings	Refs
NTN	Th2-prone BALB/c mice developed a proliferative, non-crescentic GN with renal neutrophil accumulation. Linear IgG deposition was observed in glomerular capillary loops.	[40]
	IL-4 was produced by splenocytes. BALB/c mice developed glomerular injury characterized by thickened capillary walls, mesangial expansion, and glomerular hypercellularity.	[47]
	In C57BL/6 mice, IL-4 application attenuated glomerular immune cell accumulation and crescent formation and reduced serum levels of IgG2a and IgG3.	[101]
	Lack of IL-4 in BALB/c mice did not exacerbate GN towards cGN.	[60]
Human IgAN	Imbalance of Th1/Th2 cytokines in favor of Th2 cytokines might lead to disease progression in IgAN.	[102]
	Elevated levels of abnormally glycosylated IgA were present in patients.	[105,106]
	IL-4 promoted IgA1 production and higher secretion of aberrantly glycosylated IgA1.	[107,108]
Murine IgAN	DdY mice with quiescent disease showed strong Th2 response.	[56]
	Overproduction of type 2 cytokines correlated with increased serum levels of IgA, glomerular IgA deposition, aberrantly glycosylated IgA, and proteinuria in *Smad4^co/co;Lck-cre^* mice.	[103]

**Table 3:** Th17-cell responses in human and murine cGN.

Type of GN	Key Findings	Refs
NTN	Th17 cells were detected in the kidney. IL-23p19^−/−^ and IL17A^−/−^ mice showed less crescent formation, reduced proteinuria, tubulointerstitial injury and glomerular immune cell infiltration.	[110]
	Transfer of in vitro polarized Th17 cells to Rag1^−/−^ mice led to crescent formation. Th17 cells were stable and did not start to produce IFNγ.	[87,111]
	Transfer of RORγt^−/−^ CD4^+^ T cells to Rag1^−/−^ mice resulted in reduced crescent formation.	[109]
	IL-17A^−/−^ mice were protected from NTN on day six of disease but not on day 14, and cGN was more severe on day 21 because of an enhanced Th1 response. IL-23p19^−/−^ showed more severe cGN on day 21 than WT mice.	[86]
	Renal CCL20 was upregulated and renal Th17-cell infiltration was abrogated in CCR6^−/−^ mice.	[37]
	Th17 cells showed inflammation-induced migration from the intestine to the kidney in a sphingosine-1 phosphate receptor 1 and CCL20/CCR6-mediated fashion. Renal Th17 number was influenced by the intestinal microbiome.	[120]
	Il-17F^−/−^ mice developed less severe GN associated with reduced renal neutrophil infiltration.	[121]
	Renal intrinsic cells expressed IL-17C and renal Th17 cells expressed IL-17RE.	[122]
	Lack of IL-17RC on Th17 cells aggravated GN.	[123]
	Anti-CD3 treatment induced IL-10 production in Th17 cells.	[111]
	Th17 cells converted to IL-10^+^ Tr1 cells under anti-CD3 treatment but did not seem to play a regulatory role.	[129]
	Infection with *S. aureus* induced T_RM_17 cells that persisted in the kidney after cleared infection and aggravated NTN. T_RM_17 cells were activated by Th17-polarizing cytokines.	[92]
OVA as GBM-fixed antigen in mice	Transfer of OVA-specific Th17 cells induced non-crescentic GN with renal neutrophil recruitment and enhanced renal expression of CXCL1. Th17 cells induced early tissue injury and proteinuria. Transferred Th17 cells were phenotypically stable.	[87]
NZB/NZW mice	Elevated numbers of Th17 cells correlated with accelerated LN.	[116]
MRL-*lpr* mice	Progression of LN was associated with higher expression of IL-17A and IL-23R by T cells.	[114]
	Transfer of IL-23-pre-treated lymphocytes from MRL-*lpr* mice to Rag1^−/−^ mice induced nephritis.	[114]
	Blockage of IL-23 diminished IL-17A expression and proteinuria.	[115]
Pristane-induced murine LN	Th17-cell phenotype was stable and not plastic in LN.	[111]
Human LN	Elevated serum levels of IL-17A were detected in SLE patients.	[88,90,130]
	Elevated serum levels of IL-23 were detected in SLE patients	[89,131]
	Th17 cells were determined in glomeruli and tubulointerstitium in human LN and correlated with disease activity.	[132]
	IL-17A^+^ DN T cells were detected in kidneys of LN patients.	[133]
Murine anti-MPO GN	Immunization of WT mice with MPO led to systemic IL-17A production. IL17A^−/−^ mice were protected from anti-MPO GN and showed less renal neutrophil accumulation	[112]
	Mice showed an early Th17 and a late Th1 response.	[91]
	Treatment of mice with anti-IL-23p19 Ab blocked early GN.	[91]
Human ANCA-GN	Elevated numbers of blood Th17 cells and levels of Th17 cell-associated cytokines were determined and correlated with disease activity.	[134,135]
Human IgAN	IL-17A expression was present at renal tubular sites, correlating with renal damage and impaired renal function.	[95,96]
	Enhanced numbers of circulating Th17 cells and increased serum levels of IL-17A were detected.	[94]
	Secretion of aberrantly glycosylated IgA1 was induced by IL-17A.	[136]
ddY mice	Mice showed elevated numbers of renal Th17 cells and enhanced IL-17A expression. CCL20 was upregulated and neutralization of CCL20 decreased renal Th17 cell infiltration.	[113]
Murine EAG	IL-23p19^−/−^ mice developed less severe EAG.	[28]
	Renal Th17 cell infiltration led to disease exacerbation.	[57]
	Autoreactive Th17 cells were found in EAG. IL-17A^−/−^ and IL-23p19^−/−^ mice showed less crescent formation and reduced tubulointerstitial damage.	[58]

**Table 4:** CD8^+^ T-cell responses in human and murine cGN.

Type of GN	Key Findings	Refs
NTN	CD8^+^ T-cell depletion led to reduced crescent formation, proteinuria and glomerular macrophage infiltration in rats.	[146]
	CD8^−/−^ mice developed accelerated disease.	[44]
	Transporter associated with antigen processing-deficient mice with reduced CD8^+^ T-cell numbers were not protected from NTN. CD8^+^ T-cell depletion led to reduced proteinuria and recruitment of CD4^+^ T cells and macrophages to the kidney.	[148]
	Transfer of EGFP-specific CD8^+^ T cells to mice with EGFP-expression by podocytes together with NTN induction induced severe cGN and CD8^+^ T cells infiltrated glomeruli with a ruptured BC.	[15]
Five-sixth nephrectomy	DCs, isolated from renal lymph nodes, presented albumin-derived peptides, processed through PTECs, to stimulate inflammatory CD8^+^ T-cell activation.	[151]
MRL-*lpr* mice	Renal CD8^+^ T cells were exhausted.	[155]
	Renal CD8^+^ T_RM_ cells expressed CD69 and CD103, were not exhausted and expressed GzmB, perforin, IFNγ, and TNFα and correlated with disease severity.	[156]
Human LN	Periglomerular CD8^+^ T-cell accumulation correlated with disease activity, BC rupture, and crescent formation.	[7]
	CD8^+^ T cells clonally expanded and persisted for years in the kidney.	[6]
	Cytotoxic CD8^+^ T cells were present in the kidney, either expressing GzmB and perforin or GzmK. CD8^+^ T_RM_ cells expressed Hobit, CD103, and CD49a	[143]
	CD8^+^ T_RM_ cells expressed CD103.	[156]
Human anti-MPO GN	CD8^+^ T cells infiltrated the nephritic kidney.	[4,5,6,7,142]
Murine anti-MPO GN	CD8^+^ T-cell depletion after MPO immunization attenuated segmental necrosis, proteinuria, and CD4^+^ T cell- and macrophage recruitment to glomeruli.	[149]
	Transfer of MPO-specific CD8^+^ T cells to Rag1^−/−^ mice prior to disease induction aggravated GN.	[149]
Human ANCA-GN	AAV patients with activated CD8^+^ T cells in blood had a poor prognosis.	[144,145]
Murine EAG	CD8^+^ T-cell depletion prevented EAG and ameliorated existing disease.	[147]

**Table 5:** Immunomodulators approved or in clinical development for treatment of cGN.

Biological	Disease	Approved/Clinical Development	Refs
Secukinumab (anti-IL-17A)	LN	Phase III clinical trial	[159]
Ustekinumab (anti-IL-12/IL-23-p40)	Psoriasis, psoriatic arthritis, Crohn’s disease SLE	Approved Phase II clinical trial	[162]
Belimumab (anti-BAFF)	SLE	Approved	[164]
Belimumab + rituximab (anti-CD20)	LN	Approved	[165,166]
Avacopan (C5a receptor antagonist)	ANCA-associated GN AAV, in combination with rituximab or cyclophosphamide	Pre-clinical development Approved	[167]
Low-dose IL-2	SLE, LN	Clinical development	[172,173,174,175,176,177,178,179,180]

## Data Availability

Not applicable.

## Abbreviations

ANCA: anti-neutrophil cytoplasmic antibody, APC: antigen-presenting cell, BAAF: B cell-activating factor, BC: Bowman’s capsule, cGN: crescentic glomerulonephritis, DC: dendritic cell, DN: double negative, EAG: experimental autoimmune GN, EGFP: enhanced green fluorescent protein, GBM: glomerular basement membrane, GN: glomerulonephritis, GzmB: granzyme B, Hobit: homolog of Blimp-1 in T cells, IC: immune complex, IFNγ: interferon γ, IgAN: immunoglobulin A nephropathy, LN: lupus nephritis, MHC-II: major histocompatibility complex class II, MIF: macrophage migration inhibitory factor, MPO: myeloperoxidasse, MRL-*lpr*: MRL/MpJ-*Fas^lpr^*, NTN: nephrotoxic nephritis, OVA: ovalbumin, PR3: proteinase 3, PTEC: proximal tubular epithelial cell, RPGN: rapidly progressive GN, SLE: systemic lupus erythematosus, TCR: T-cell receptor, Th: T helper, TNFR1: tumor necrosis factor receptor 1, TNFα: tumor necrosis factor α, T_RM_: tissue-resident memory, WT: wild type.
